# Management of Buccal Screw Access Hole Positioning for Implant Fixed Complete Dentures: A Report of Two Patients and a Proposed Decision Tree

**DOI:** 10.1155/2021/4279764

**Published:** 2021-12-21

**Authors:** Fawaz Alzoubi

**Affiliations:** Department of General Dental Practice, Faculty of Dentistry, Kuwait University, Jabriya, Kuwait

## Abstract

The present report demonstrated the use of two methods to correct the positioning of buccal screw access holes in both implant- and abutment-level implant-supported fixed complete dentures in two patients. The report suggests that nonaxially tightened abutments (in this report, dynamic abutments), angled multi-unit abutments, and the use of a milled framework with individual crowns aid in masking buccal screw access holes while maintaining the benefits of a screw-retained design. A decision tree is also proposed to facilitate the workflow when faced with such clinical scenarios.

## 1. Introduction

Implant fixed complete dentures (IFCDs) are a well-documented, predictable, and reliable treatment method [1–4]. Nevertheless, the installation of IFCDs is a complex procedure, and issues related to its execution are common. One of the primary issues encountered involves the buccal emergence of screw access holes (SAHs).

Screw-retained implant prostheses are generally preferred because of their advantageous design, especially in relation to retrievability [5–8]. However, an esthetic problem arises in cases where the implant trajectory results in a buccally positioned SAH; hence, a cement-retained design may be used to mask the buccal SAH [9]. Although this technique might be acceptable for partially edentulous cases, it is generally not recommended to have cement-retained designs in IFCDs [9]. The reasons to avoid this technique include an extreme difficulty in retrieving the prosthesis without damaging it [10], increased likelihood of having remnant cement around the implants, potentially causing peri-implant disease [11–14], and a high chance of cement setting before the complete seating of the prosthesis, leading to possible misfit.

During the retrieval of a cement-retained IFCD, it is very likely to damage the prosthesis while accessing the prosthetic screws, a scenario that not only requires a considerable amount of time and effort but also carries a financial burden because of the likelihood of having to reexecute the entire prosthetic treatment. Removing excess cement from single or partial implant-supported fixed dental prostheses [14] is a challenging procedure because of the limited accessibility to the peri-implant environment, which would be even more complicated if it was executed for a full-arch prosthesis including four to eight implants. In case of IFCDs, a passive fitting (or minimal misfit) of the prosthesis is desired in order to minimize any biomechanical complications and possible failure [15–18]. With a cement-retained IFCD, the clinician must ensure that the cement is mixed and applied evenly throughout multiple implant abutments. This constitutes a significant challenge because the cement is likely to start setting on the first abutment before the clinician is able to apply any of it on the last abutment. This difference in application and setting time impedes the complete seating of the prosthesis because of the hard consistency of the cement that begins to set in the abutments where it was first applied, hence impeding passive seating of the framework and increasing the likelihood of misfit and biomechanical problems.

The literature currently reports a great understanding of several factors related to the proper installation of IFCDs. However, authors are inclined to report technical methods to address any potential obstacles (such as positioning of buccal SAHs) to assist colleagues in managing these scenarios and optimize implant-based treatment involving buccal SAH, which is a current challenge in the field of dentistry. Hence, the aim of this paper is to describe two methods for managing the positioning of buccal SAHs for IFCDs by reporting two clinical cases and proposing a decision tree to facilitate clinicians' workflow when faced with similar scenarios. This may have significant clinical implications, as it will aid clinicians in making informed and appropriate clinical decisions in this type of scenario.

## 2. Case Presentations

### 2.1. Case One

A 53-year-old female patient presented to the clinic requesting replacement of her missing and compromised teeth with a fixed prosthesis. Medical history was reviewed and considered noncontributory. After clinical and radiographic examinations, the proposed treatment was a maxillary IFCD supported by five implants. Five 4.5 × 11 mm implants (NobelReplace Conical Connection, Nobel Biocare, Kloten, Switzerland) were placed as planned, and an immediate removable complete denture (RCD) was relined and delivered after surgery. This approach was selected because of the sufficient availability of bone on the maxillary right side and the lack of bone on the maxillary left molar side. To avoid a sinus augmentation procedure, the left maxillary posterior implant was tilted, and the trajectory was corrected using a 30° multi-unit abutment.

### 2.2. Case Two

A 70-year-old female patient presented to the clinic requesting replacement of her missing mandibular teeth with a fixed prosthesis. Medical history was significant for diabetes mellitus, hypertension, hyperlipidemia, hypothyroidism, benign positional vertigo, anemia, and nonalcoholic steatohepatitis. The patient used multiple medications to control her underlying conditions. After clinical and radiographic examinations, the proposed treatment was a mandibular IFCD supported by four implants using an All-on-4 approach. Two 5 mm × 10 mm tilted implants (NobelReplace Conical Connection, Nobel Biocare, Kloten, Switzerland) were placed in the posterior sites using 30° multi-unit abutments, and two 4.3 mm × 10 mm axial implants (NobelReplace Conical Connection, Nobel Biocare, Kloten, Switzerland) were placed in the anterior sites using straight multi-unit abutments. An immediate RCD was relined and delivered after surgery. This approach was selected because of the lack of bone in the area posterior to the mental foramina and to avoid considerable bone grafting procedures in an already medically compromised patient.

### 2.3. Clinical Outcomes

Fixed implant rehabilitation was selected for both patients to enhance quality of life by improving mastication, esthetics, and psychological well-being, since both patients could not tolerate removable prostheses. Furthermore, in both cases, resin-template guides were produced after duplicating the respective complete dentures and were used during surgery to guide the initial drills into the planned osteotomy sites.

**Figure 1:**
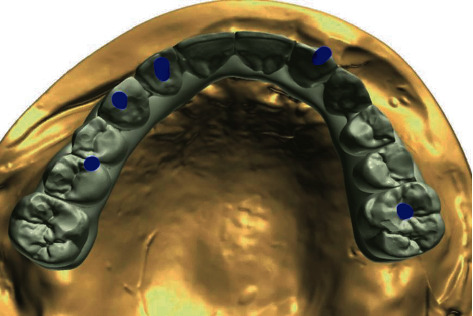
Digital superimpositions of the tooth setup and implant master cast scans for case one indicating buccal SAH on site 22 and insufficient thickness of buccal material in site 13.

**Figure 2:**
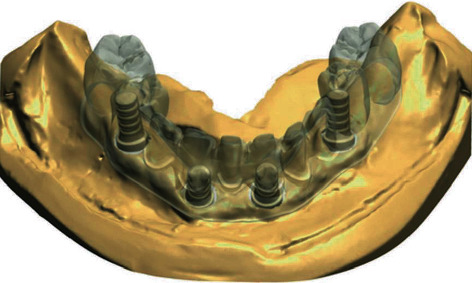
Digital superimpositions of the tooth setup and implant master cast scans for case two indicating buccal SAHs in the two anterior implants.

**Figure 3:**
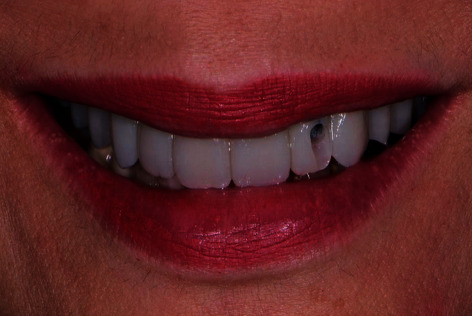
Case one: maximum smile with provisional implant showing visibility of the buccal SAH.

**Figure 4:**
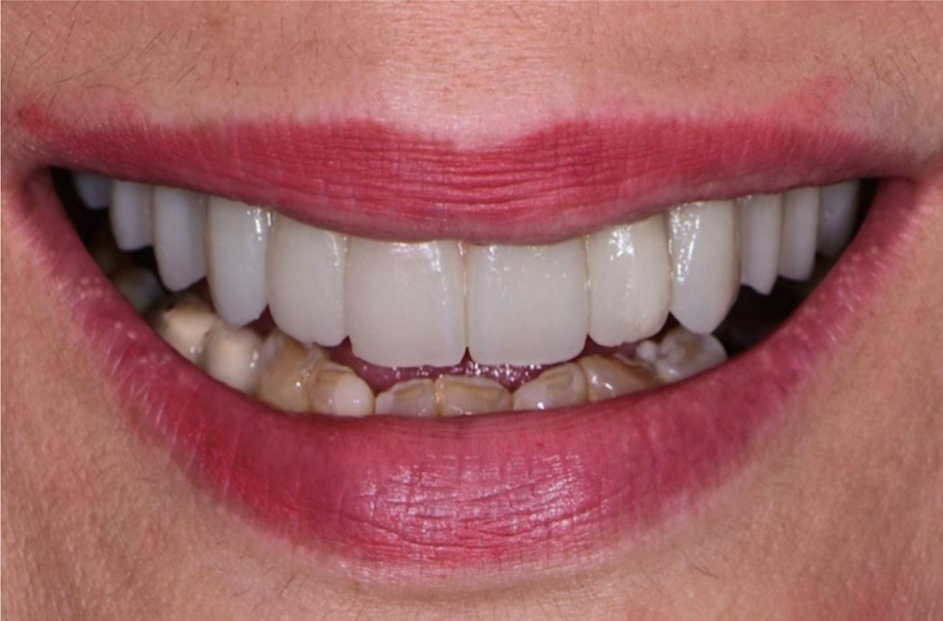
Case one: maximum smile after masking with resin.

**Figure 5:**
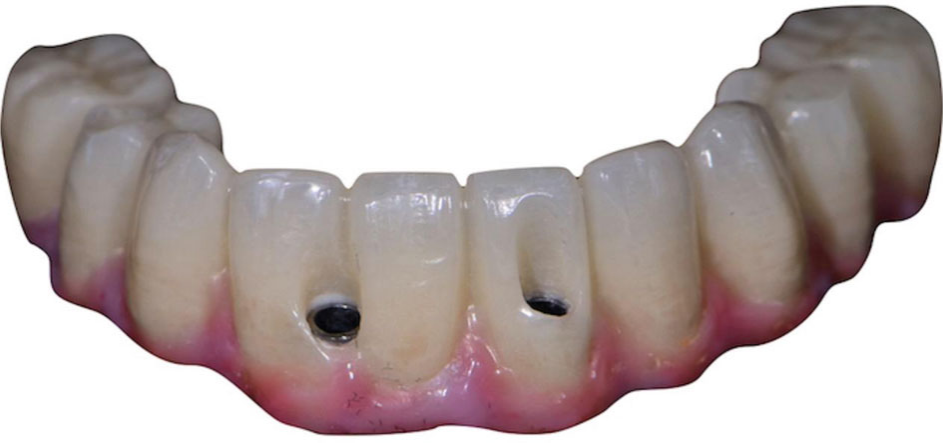
Provisional IFCD with buccal SAH in case two.

Both patients were followed up after approximately 4 months of uneventful healing. Temporary milled polymethyl methacrylate- (PMMA-) provisional IFCDs were fabricated. In both patients, it was noticed during the digital design phase of the provisional IFCDs that the implant trajectories of some anterior implants led to buccally positioned SAHs. Furthermore, in case one, the implant trajectory at site 13 (maxillary right canine) did not present sufficient thickness of material buccal to a the SAH (Figures 1 and 2). Nonetheless, the lab was instructed to proceed with fabricating screw-retained, PMMA-provisional IFCDs with an understanding of the aforementioned issues. Both provisional IFCDs were delivered, and the buccal SAHs were masked with resin in an attempt to match the tooth shade as accurately as possible (Figures 3, 4, and 5). After approximately 8 weeks, patients were recalled for evaluation and did not report any significant complaints; however, it was noticed that the resin masking the buccal SAHs was stained, compromising the esthetic result.

**Figure 6:**
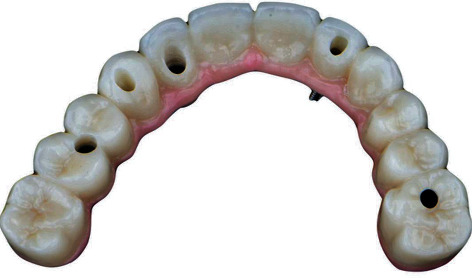
Case one: occlusal view of the final IFCD demonstrating lingual SAH on site 22 and improved thickness of buccal material in site 13.

**Figure 7:**
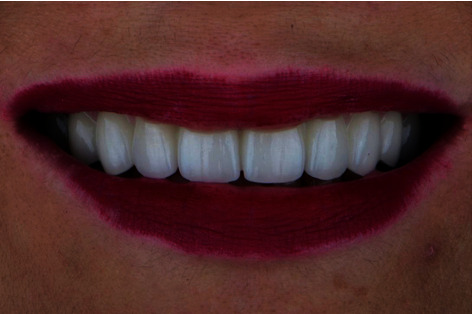
Case one: maximum smile after installation of the final IFCD with lingualized SAH using DA.

For case one, nonaxially tightened dynamic abutments (DA) were used to correct the buccal SAH in implant 22 (maxillary left lateral incisor) and lingualize the SAH in implant 13 to allow for increased thickness of the prosthetic material that was buccal to the SAH. Finally, a combination of an implant- and abutment-level (only on the left maxillary posterior implant) splinted IFCD with a milled zirconia framework, which was layered with lithium disilicate on the anterior teeth, was fabricated and delivered (Figures 6 and 7).

**Figure 8:**
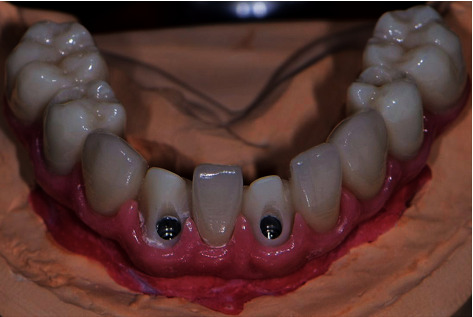
Final milled zirconia framework with individual crowns in case two: abutments milled for anterior teeth with buccal SAH.

**Figure 9:**
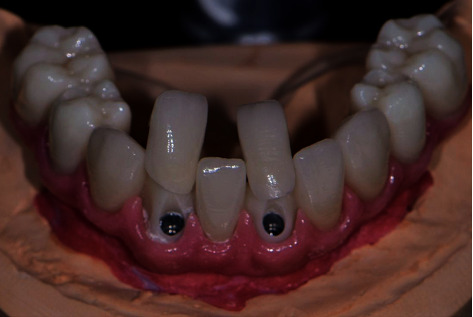
Final milled zirconia framework with individual crowns in case two: individual *E*max crowns fabricated to be cemented intraorally.

**Figure 10:**
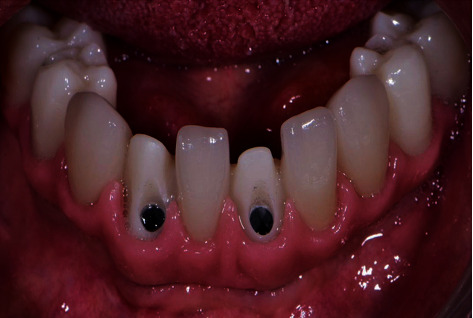
Final milled zirconia framework with individual crowns in case two: after securing the implants and torquing the prosthetic screws.

**Figure 11:**
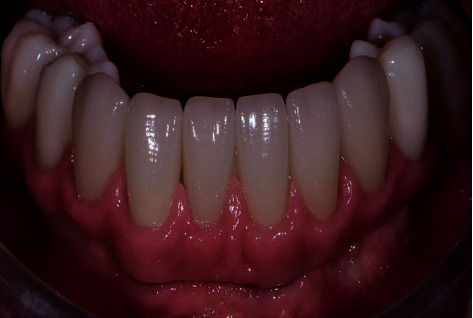
Final milled zirconia framework with individual crowns in case two: after intraoral *e*.max crown cementation.

For case two, to correct the buccal SAHs in the anterior two implants, the lab was instructed to fabricate an abutment-level splinted milled zirconia framework with individual lithium disilicate crowns for the buccal SAH sites (Figures 8 and 9). This design lends itself to a screw-retained approach and masks buccal SAHs via the intraoral cementation of individual crowns. The prosthesis was then delivered, screws were torqued according to manufacturer's recommendations, and individual crowns were cemented with resin only on the sites presenting buccal SAHs (Figures 10 and 11).

## 3. Discussion

Although implant placement should be prosthetically driven and placed to accommodate a screw-retained position, bone topography and quantity might obstruct such placement, especially in the anterior region. Bone augmentation may be recommended; however, in completely edentulous cases, this procedure might be challenging and present questionable prognosis due to factors such as age (older age is often accompanied by medically compromised health) and the amount of augmentation necessary to regain lost bone. Hence, interest has been invested into prosthetic solutions to overcome these surgical limitations [19–23], such as DAs and angled multiunit abutments.

DAs allow for screw-retained designs by using a titanium screw that is torqued in a nonaxial manner [22], permitting angular correction by up to 28° [24]. In addition, a significant advantage is that an implant-level prosthesis can be fabricated. Hence, there is no need for additional restorative components to correct for angulation (e.g., angled multi-unit abutments), which might compromise available space and esthetic outcomes, especially when there is not sufficient soft tissue around the implant (such as in case one). If an angled multiunit abutment was used to lingualize implant trajectories at sites 13 and 22, then not only would the metal collar show, leading to an esthetic issue, but it would also compromise the already-limited prosthetic space available. A considerable limitation of DAs is that the screw torque values required to secure the abutments to the implant are generally lower than conventional values; hence, it is plausible to hypothesize the subsequent occurrence of issues involving screw loosening. Goldberg et al. [22] concluded that the removal torque and fatigue strength of DAs were comparable to those of gold screws and that the angulation of the abutment had no significant influence on screw removal torque values. However, Swamidass et al. [23] concluded that torque values presented greater torque differences in angulated access channel crowns. Although this is still a promising method for the angulation correction of implant trajectory, the literature lacks strong evidence to support its long-term clinical viability, especially in completely edentulous patients.

In case two, an All-on-4 approach was used, with angled multi-unit abutments for the posterior implants and with straight multiunit abutments for the anterior implants. It was noticed during the digital design of the provisional IFCDs that the implant trajectories for the anterior two implants led to the buccal placement of SAHs. A solution for this could have been to retrieve the straight multi-unit abutments and replace them with angled multi-unit abutments to lingualize the SAH. Nonetheless, this procedure might have interrupted the established soft tissue seal between the abutment and gingiva, leading to potentially negative effects on soft tissue and marginal bone levels [25]. In addition, this option was not feasible as we strived to minimize the number of visits because patient's older age and complex medical history implied significant physical restrictions; indeed, if the multi-unit abutments were replaced, the prosthetic workflow would need to be reexecuted, involving three or four additional visits. Therefore, the author opted to use an abutment-level splinted milled zirconia framework with individual lithium disilicate crowns in buccal SAH sites. Although this approach aids in masking buccal SAHs, the crowns in the respective sites are damaged in the process of prosthesis retrieval. However, the extent of damage is limited to the two crowns, which can be easily replaced in the future, instead of damaging the entire prosthesis.

The use of milled frameworks with individual crown designs has gained popularity for several reasons. In this paper, the author recommends the use of this option for abutment-level or implant-level prostheses; however, as previously mentioned, only if the screw access channel (SAC) trajectory leads to a buccally positioned SAH in individual sites. The proposed solution includes a framework that can be milled for a screw-retained implant; in sites where the SAH is positioned buccally, the lab can be instructed to design the framework in a way that can accommodate individual crowns by digitally designing and milling the abutments accordingly. Then, the fabricated individual crowns are to be cemented in the mouth after the delivery of the screw-retained prosthesis. Hence, masking the SAHs still results in a screw-retained prosthesis. If the prosthesis needs to be retrieved in the future, then only those sites' crowns are damaged, which can then be individually replaced, and the remaining prosthesis can be retrieved by accessing the screws through the SAHs. Furthermore, this method can be used if DAs cannot be used due to a needed angulation correction exceeding 28°.

**Figure 12:**
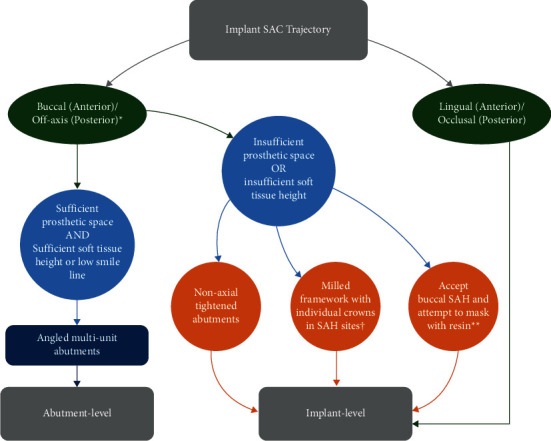
Proposed decision tree to facilitate the management of buccal SAH positioning. SAC: screw access channel; SAH: screw access hole. ^∗^Including tilted implants; ^∗∗^only recommended for provisional prostheses or cases where the SAH is masked by the lip line during maximum smiling; ^†^can also be performed for abutment-level prostheses. In addition, can be used if DAs cannot be used due to a needed angulation correction exceeding 28°.

Regarding the masking of buccal SAHs, various resin materials may be used; however, potential staining at the sites of interest is to be expected. Hence, the author recommends using resin materials strictly in the case of provisional restorations or when the SAH is masked by the lips during maximum smiling. Based on the presented report, a decision tree is proposed to facilitate the clinical decision-making process when faced with similar scenarios (Figure 12).
